# Comparative genome analysis of the *Lactobacillus brevis* species

**DOI:** 10.1186/s12864-019-5783-1

**Published:** 2019-05-23

**Authors:** Marine Feyereisen, Jennifer Mahony, Philip Kelleher, Richard John Roberts, Tadhg O’Sullivan, Jan-Maarten A. Geertman, Douwe van Sinderen

**Affiliations:** 1 School of Microbiology, University College Cork, Cork, Ireland; 2 APC Microbiome Ireland, University College Cork, Cork, Ireland; 3 New England BioLabs, Inc., Ipswich, MA USA; 4 HEINEKEN Global Supply Chain B.V, Zoeterwoude, The Netherlands

**Keywords:** *Lactobacillus brevis*, SMRT sequencing, Genomics, Pan-genome, Beer adaptation, Beer spoilage

## Abstract

**Background:**

*Lactobacillus brevis* is a member of the lactic acid bacteria (LAB), and strains of *L. brevis* have been isolated from silage, as well as from fermented cabbage and other fermented foods. However, this bacterium is also commonly associated with bacterial spoilage of beer.

**Results:**

In the current study, complete genome sequences of six isolated *L. brevis* strains were determined. Five of these *L. brevis* strains were isolated from beer (three isolates) or the brewing environment (two isolates), and were characterized as beer-spoilers or non-beer spoilers, respectively, while the sixth isolate had previously been isolated from silage. The genomic features of 19 *L. brevis* strains, encompassing the six *L. brevis* strains described in this study and thirteen *L. brevis* strains for which complete genome sequences were available in public databases, were analyzed with particular attention to evolutionary aspects and adaptation to beer.

**Conclusions:**

Comparative genomic analysis highlighted evolution of the taxon allowing niche colonization, notably adaptation to the beer environment, with approximately 50 chromosomal genes acquired by *L. brevis* beer-spoiler strains representing approximately 2% of their total chromosomal genetic content. These genes primarily encode proteins that are putatively involved in oxidation-reduction reactions, transcription regulation or membrane transport, functions that may be crucial to survive the harsh conditions associated with beer. The study emphasized the role of plasmids in beer spoilage with a number of unique genes identified among *L. brevis* beer-spoiler strains.

## Background

*Lactobacillus brevis* is a member of the lactic acid bacteria (LAB), which are catalase-negative, non-sporulating, non-motile, rod or coccus-shaped Gram-positive bacteria. *L. brevis* grows optimally at 30 °C and within a pH range of 4 to 6 [1–3]. It is an obligatory hetero-fermentative bacterium producing lactic acid, carbon dioxide and ethanol and/or acetic acid [1–3]. Using phylogenomic and comparative genomic analysis Duar et al. studied the relatedness within the *Lactobacillus* genus in light of their natural habitat in order to understand their evolutionary history [4]. They assigned lactobacilli species into three main lifestyle categories: free living (environmental and plant isolates), host adapted or as “nomadic” [4]. Sequenced genomes of the *Lactobacillus* genus range in size from 1.27 (*L. iners*) to 4.91 (*L. parakefiri*) Mbp [4].

*L. brevis* has been isolated from silage, as well as from fermented cabbage and other fermented foods [5, 6], and is assigned to the free-living lifestyle group of lactobacilli [4]. *L. brevis* strains, among other lactobacilli, are of particular interest as they have been granted Qualified Presumption of Safety (QPS) status and consequently have been widely used in the production of fermented foods [1, 7]. In addition to their application in food fermentations they are purported to have potential as health-promoting or probiotic bacteria [1, 7]. In contrast to these positive attributes, *L. brevis* strains have also been reported as the causative agent of food or beverage spoilage, in particular of beer [8, 9]. LAB species are reported to cause approximately 70% of microbial beer-spoilage incidents, and among this group *L. brevis* isolates are particularly problematic [10–12]. They are associated with the production of malodorous compounds, acidity and/or turbidity with negative impacts on the organoleptic properties of the final product. Bacterial spoilage of beer may result in product withdrawal or recall with concomitant economic losses for the brewing industry [10–12]. Beer spoilage by Gram-positive bacteria has been studied previously and the main mechanism of hop resistance known so far involves an active extrusion of the toxic compound using transporters identified as: (a) HorA which functions as an ABC-type multidrug transporter to expel hop compounds, in particular *iso-α-*acids, from the bacterial cytoplasm, (b) HorC a proton motive force-dependent hop excretion transporter, and (c) the H^+^-ATPase which increases the pumping of protons released from the hop compounds [13–15]. The transmembrane protein HitA is also thought to play a role in the transport of divalent cations, where iso-α-acids exchange protons for cellular divalent cations such as Mn^2+^ [16].

To date a number of comparative genome studies of the *Lactobacillus* genus have been described [1, 17–19], some of which have provided insights into the taxonomy of the *Lactobacillus* genus [3, 7], or its fermentation capabilities [3]. Carbohydrate metabolism has been assessed in several *Lactobacillus* species LAB such as *L. casei* or *L. plantarum* [2]. However, a broad comparative genome analysis of the *L. brevis* species has as yet not been undertaken. Recent advances in next generation sequencing technologies has facilitated a rapid surge in the number of bacterial genomes now available for comparative analysis within a genus or a species.

In the current study, Single-Molecule-Real-Time (SMRT) sequencing technology [20, 21] was employed to generate the complete genome sequence of an additional six *L. brevis* strains isolated from silage and the brewery environment. Using the dataset of 19 complete chromosomal sequences, a comparative genome analysis of the *L. brevis* taxon was undertaken through an assessment of the phylogeny, pan- and core-genome, and niche adaptation with particular emphasis on adaptation to the brewing environment. The importance of plasmids was also investigated in relation to beer spoilage ability.

## Results and discussion

### Isolation of *L. brevis* strains

**Fig. 1:**
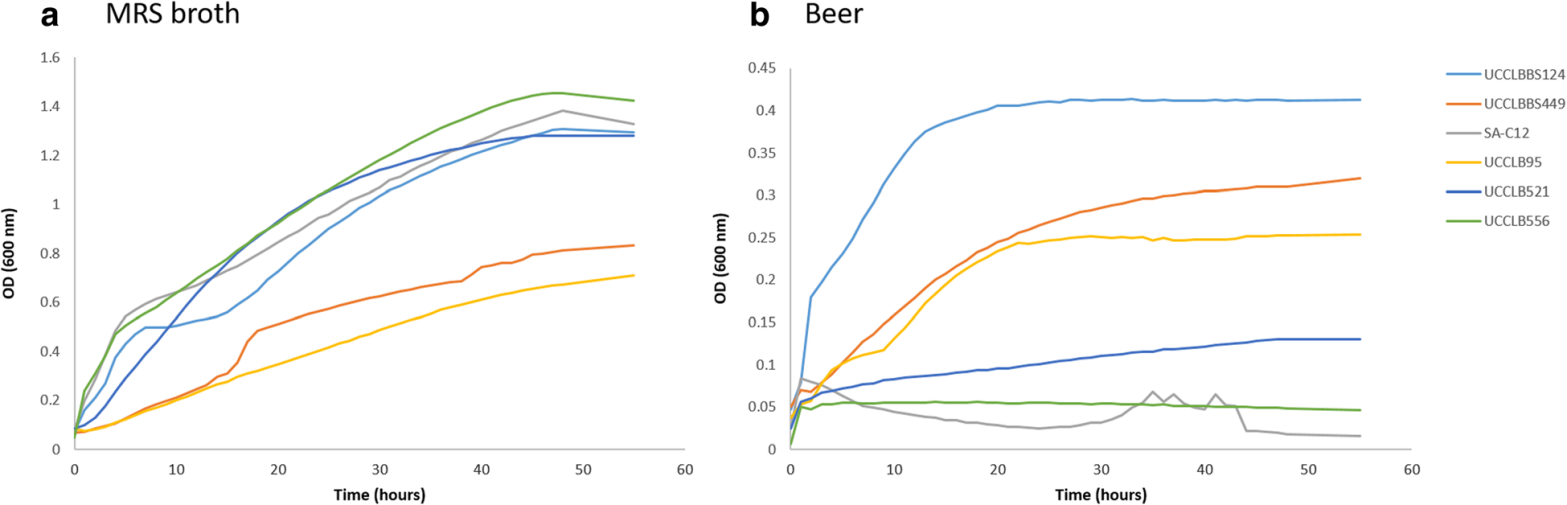
Growth profile of *L. brevis* strains sequenced in this study. Growth profile of *L. brevis* strains UCCLBBS124, UCCLBBS449, UCCLB95, UCCLB521, UCCLB556 and SA-C12 in (**a**) MRS broth or (**b**) beer. Growth curves were performed in triplicate and the average of those measurements is displayed in the graph above

Six *L. brevis* strains were isolated and included as part of the study, with the aim of expanding the collection of *L. brevis* genome sequences currently available, as well as studying their ability to grow and colonize harsh environments such as beer. Three *L. brevis* strains were isolated from beer and characterized as beer-spoilers based on their ability to grow in beer. Two additional *L. brevis* strains were isolated from the brewing environment, yet lack the ability to grow in beer and are thus not classified as beer-spoilers (Table 1 and Fig. 1). In addition, the sixth *L. brevis* strain sequenced as part of this study originates from silage [22] and was included as a non-brewing environmental isolate (Table 1). The different *L. brevis* isolates showed different colony morphology ranging from a dry irregular colony type for *L. brevis* UCCLBBS449 and UCCLB95 to a slimy and ropy colony type for UCCLBBS124 (Table 1). Plasmid profiling of the different isolates revealed a distinct plasmid content for each isolate. Growth curves in MRS broth demonstrated the unique growth profiles of the individual isolates confirming that the isolates were distinct from each other. Furthermore, only *L. brevis* UCCLB95, UCCLBBS124 and UCCLBBS449 were characterized as beer-spoilers having the ability to survive and grow in beer, while *L. brevis* SA-C12, UCCLB521 and UCCLB556 were defined as non-beer-spoilers (Table 1 and Fig. 1).

**Table 1:** Isolation of *L. brevis* strains

Isolation source	*L. brevis*	Colony morphology	Plasmids	Ability to grow in beer	Spoiled beer characteristics
Silage	SA-C12	Rounded	2	No	N/A
Brewery	UCCLB521	Rounded	5	No	N/A
Brewery	UCCLB556	Rounded	7	No	N/A
Beer	UCCLB95	Dry, irregular edges	2	Yes	Turbid
Beer	UCCLBBS124	Slimy, ropy, rounded	4	Yes	Slimy, ropy, turbid
Beer	UCCLBBS449	Dry, irregular edges	9	Yes	Turbid

N/A not applicable

### General genome features

The complete chromosomal sequences of nineteen *L. brevis* strains were selected for analysis, thirteen of which available at that time were obtained from the NCBI database, while the remaining six were sequenced as part of this study using SMRT sequencing technology (Table 2). These 19 selected *L. brevis* strains had been isolated from different ecological niches: silage, fermented food, animal’s gut and the brewery environment (Table 2). The general features of the 19 *L. brevis* genomes are indicated in Table 3 and include an average chromosome length of 2.49 Mbp (ranging from 2.27 to 2.79 Mbp) and a G + C content of 46%. An average of 2338 predicted CDSs (Coding Sequences) per chromosome were identified to which approximately 78.3% could be assigned a function based on in silico predictions using BLAST (Basic Alignment Search Tool), while the remaining 21.7% were annotated as hypothetical proteins (Table 3). A type II CRISPR-Cas (Clustered Regularly Interspaced Short Palindromic Repeats) locus was found in the chromosome of *L. brevis* BGP6, *L. brevis* NPS-QW145 and *L. brevis* SRCM101106 where variability was observed in the spacer region, distinct spacers were observed in each of these three *L. brevis* strains suggesting an active system acquiring unique and various spacers for protection against invading DNA over time. Conversely, in the chromosome of *L. brevis* TMW1.2112 and *L. brevis* TMW1.2113 ten identical spacers were detected suggesting that these two strains are clonal or that this CRISPR-Cas system is inactive, and that these common spacers originate from a common ancestor that acquired genetic material from viruses/plasmids that it encountered in the past [23]. The *L. brevis* strain ZLB004 chromosome revealed the presence of four CRISPR locus, one was associated to a type I-E CRISPR-Cas system, a second one was associated to a type II CRISPR-Cas system both potentially active systems. The two other CRISPR locus were not associated to any CRISPR-Cas proteins suggesting inactive system.

**Table 2:** *Lactobacillus brevis* strains and/or genomes used in this study

Strain name	Genbank accession	Ecological niche	Year	Citation
100D8	CP015338	Rye silage (South Korea)	2016	
ATCC 367	CP000416	Sourdough/Silage starter culture	2006	[5]
BDGP6	CP024635	*Drosophila melanogaster* female gut	2015	
KB290	AP012167	Suguki (fermented vegetable)	2013	[49]
NPS-QW-145	CP015398	Traditional Korean Kimchi (Hong-Kong)	2016	[50]
NCTC13768	LS483405	Unknown		
SA-C12	CP031185	Silage (Ireland)	2008	[22]
SRCM101106	CP021674	Food (South Korea)	2017	
SRCM101174	CP021479	Food (South Korea)	2017	
TMW 1.2108	CP019734	Wheat beer (Germany)	2016	
TMW 1.2111	CP019743	Wheat beer (Germany)	2016	
TMW 1.2112	CP016797	Wheat beer (Germany)	2016	
TMW 1.2113	CP019750	Brewery-associated surface (Germany)	2016	
UCCLB521	CP031208	Brewery environment (The Netherlands)	2013	This study
UCCLB556	CP031174	Brewery environment (The Netherlands)	2014	This study
UCCLB95	CP031182	Beer (The Netherlands)	2001	This study
UCCLBBS124	CP031169	Beer keg (Singapore)	2003	This study
UCCLBBS449	CP031198	Unpasteurised beer (The Netherlands)	1994	This study
ZLB004	CP021456	Pig’s feces	2010

**Table 3:** General chromosomal features and plasmid content among *L. brevis* strains

*L. brevis* strain	Chromosome length (Mbp)	CDS	tRNA features	rRNA features	Hypothetical proteins %	Assigned function %	IS elements/ transposases	Prophage	CRISPR	GC %	Plasmids (Ranging size Kb)
100D8	2.35	2228	66	15	21.2	78.8	25	1 In^a^ 3 Pa^b^	–	46.1	3 (39.9–45.1)
ATCC 367	2.29	2133	65	15	20.8	79.2	34	1 In	–	46.2	2 (13.4–35.6)
BDGP6	2.79	2674	71	15	23.1	76.9	24	4 In 3 Pa	1	46.6	–
KB290	2.40	2308	64	15	21.4	78.6	50	2 In 2 Pa	–	46.1	9 (5.9–42.4)
NCTC13768	2.49	2413	65	15	15.0	85.0	3	1 Pa	–	46.0	–
NPS-QW-145	2.55	2406	62	13	21.5	78.5	5	3 Pa	1	45.8	–
SA-C12	2.44	2344	66	15	23.2	76.7	42	2 In 3 Pa	–	45.9	2 (24.8–43.6)
SRCM101106	2.44	2379	67	15	23.0	77.0	46	3 In 4 Pa	1	45.9	4 (16.0–36.2)
SRCM101174	2.41	2353	68	15	24.0	76.0	37	3 In 2 Pa	–	46.1	5 (9.4–50.4)
TMW 1.2108	2.57	2448	66	15	22.8	77.2	17	2 In	–	45.8	8 (5.1–107.0)
TMW 1.2111	2.57	2458	66	15	21.8	78.2	22	2 In	–	45.8	6 (8.2–107.0)
TMW 1.2112	2.49	2283	65	15	19.6	80.4	29	1 In 1 Pa	1	46.0	5 (8.5–59.7)
TMW 1.2113	2.54	2376	69	15	22.5	77.5	30	2 In	1	45.9	4 (8.5–46.6)
UCCLB521	2.27	2088	62	15	20.0	80.0	32	2 Pa	–	46.3	5 (11.3–43.8)
UCCLB556	2.38	2201	66	18	22.8	77.2	32	1 Pa	–	46.1	7 (4.3–68.4)
UCCLB95	2.51	2283	65	15	22.7	77.3	132	1 In 1 Pa	–	45.9	2 (3.5–14.0)
UCCLBBS124	2.61	2442	66	15	21.8	78.2	60	1 In 2 Pa	–	45.8	4 (21.0–49.6)
UCCLBBS449	2.58	2404	66	15	21.1	78.9	114	1 In 3 Pa	–	45.8	9 (2.8–66.8)
ZLB004	2.66	2207	64	15	24.0	76.0	29	1 In	2	46.0	5 (16.7–78.1)
Average	2.49	2338	66	15	21.7	78.3	40	1.4 In 1.6 Pa	–	46.0	5

^a^In: Complete intact prophage ^b^Pa: Partial/incomplete prophage

PacBio SMRT sequencing was used to determine the diversity and frequency of methylated motifs recognized by Restriction Modification (R/M) systems within the six newly sequenced and annotated *L. brevis* strains as part of this study. R/M systems constitute one of the biological barriers exerted by a strain against foreign DNA [24]. This analysis revealed the presence of various m6A motifs and allowed the identification of three motifs assignable to Type I R/M system and six motifs assignable to Type II R/M system (Table 4). The presence of specific methylated motifs was linked to the presence of specific R/M systems in the corresponding *L. brevis* strains (Table 4). Somewhat surprisingly, *L. brevis* UCCLB95 does not appear to encode any R/M systems.

**Table 4:** *L. brevis* methyltransferases with their assigned recognition sequence

*L. brevis* strain	Enzyme	Recognition sequence/motif	R/M type
UCCLBBS124	Lbr124II	CATCN**A**C	II
	M.Lbr124I	YTC**A**(N7)**T**TRG	I
UCCLB521	M.Lbr521I	**A**GG(N6)**T**TC	I
	Not assigned	G**A**TC	II
UCCLB556	M.Lbr556I	RTC**A**(N9)**T**CC	I
UCCLBBS449	Lbr449I	AGCC**A**G	II
	Not assigned	CTTGC**A**	II
UCCLB95	None detected		
SA-C12	M1.LbrSAC12IP	G**A**GGC	II
	M2.LbrSAC12I	G**A**GGC	II

Bold: m6A

### The predicted mobilome of *L. brevis*

All complete chromosome sequences were analyzed for the presence of mobile elements such as IS elements and genes specified as encoding transposases. This analysis indicates that *L. brevis* strains UCCLBBS449 and UCCLB95 contain the highest number of insertion sequence (IS) elements/transposases, 114 and 132, respectively (Table 3). The genome sequences were also investigated for prophages, revealing various predicted intact or partial prophage regions (Table 3), displaying in most cases similarity to the published *L. brevis* temperate bacteriophage LBR48 [25]. The plasmid content of the *L. brevis* strains is detailed below.

### Phylogenetic analysis

**Fig. 2:**
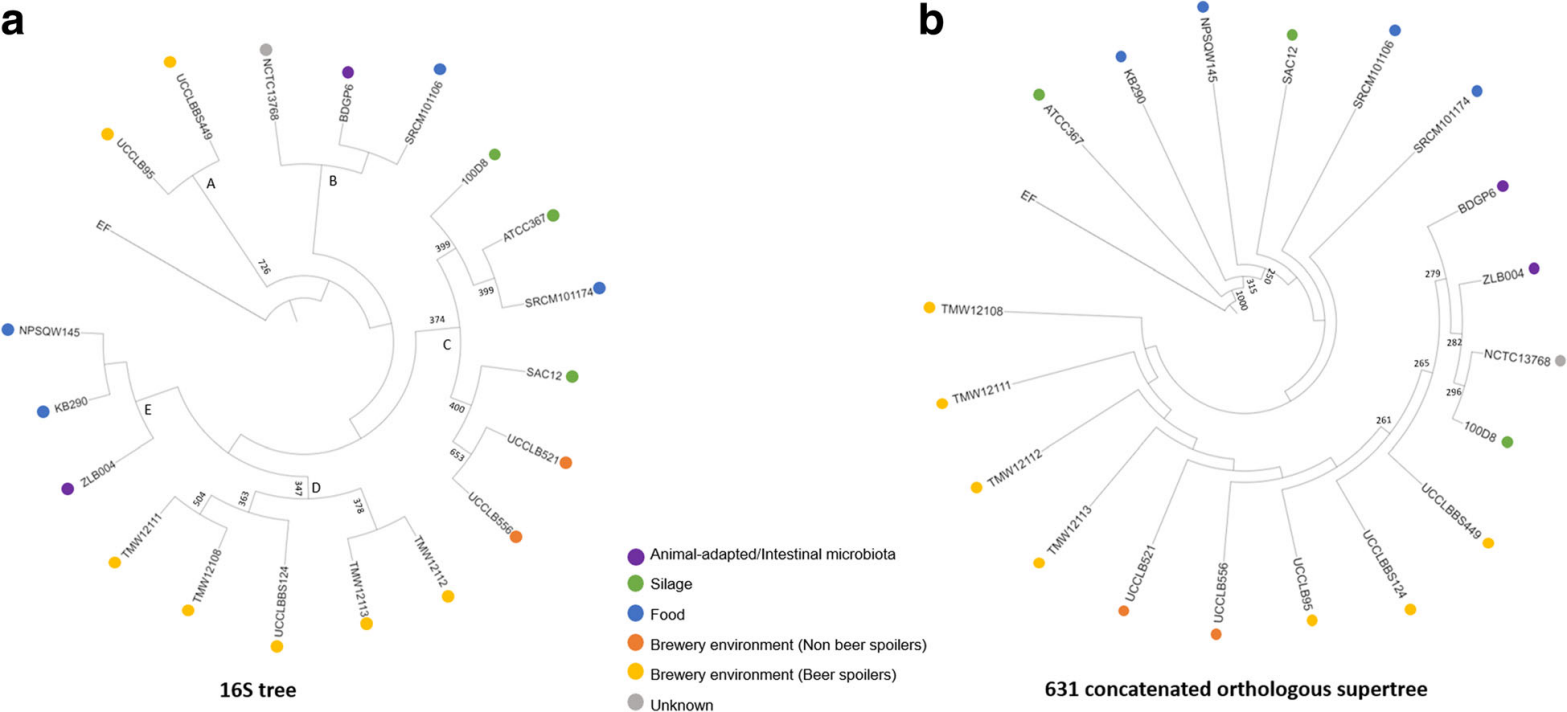
Phylogenetic analysis of *L. brevis* species. **a** 16S ribosomal tree obtained from the alignment of the 16S rRNA-encoding genes of 19 *L. brevis* strains, bootstrapped × 1000 replicates, values > 250 are indicated. The 16S rRNA sequence of *Enterococcus faecalis* V583 (noted EF on the figure) was used as an outgroup. **b** Phylogenetic supertree obtained from the alignment of 631 orthologous genes among the 19 *L. brevis* strains used in this study as well as in *Enterococcus faecalis* V583 (noted EF on the figure) which was used as an outgroup, bootstrapped × 1000 replicates, values > 250 are indicated. Source of isolation for the different *L. brevis* strains are also indicated

The phylogenetic relationship between the genomes of the nineteen *L. brevis* strains were investigated by a comparative analysis of their 16S rRNA sequences (Fig. 2a). The resulting phylogenetic tree distinguishes five clades (clades A through to E). Clade A represents two *L. brevis* beer-spoiling strains UCCLB95 and UCCLBBS449 both isolated from spoiled beer, displaying slow growth in nutritive media or beer (.

Table 1 and Fig. 1). Clade B encompasses three *L. brevis* strains: *L. brevis* SRCM101106 isolated from food *L. brevis* BDGP6 isolated from the gut of a drosophila and *L. brevis* NCTC13768 from an unknown isolation source. Clade C is represented by six *L. brevis* strains, of which one was isolated from food (*L. brevis* SRCM101174), three from silage (*L. brevis* SA-C12, ATCC 367 and 100D8) and two strains (*L. brevis* UCCLB521 and UCCLB556), both isolated from the brewing environment, yet unable to survive and grow in beer (Table 1). These latter two strains may have been introduced into the brewery through raw materials such as cereal grains thus explaining the observed phylogenetic relation to the silage *L. brevis* isolate SA-C12. Clade D includes five *L. brevis* strains, all retrieved as beer-spoiler strains from the brewing environment, and all exhibiting a slimy, ropy phenotype (Table 1) [9]. Clade E gathers three *L. brevis* strains, two isolated from fermented food (*L. brevis* KB290 and NPS-QW-145) as well as *L. brevis* ZLB004 isolated from pig’s feces.

In order to obtain a more refined view of the phylogeny of the 19 analyzed strains, a so-called phylogenetic supertree was constructed based on 631 conserved orthologous proteins that had been identified as single-copy genes conserved across all investigated chromosomal sequences (19 *L. brevis* strains and *Enterococcus faecalis* V583 as an outgroup) [26, 27]. This supertree does not display distinct clades separating the *L. brevis* strains in different groups as was observed with the 16S rRNA phylogenetic tree, suggesting a close relatedness within the species (Fig. 2b). However, upon close inspection of this phylogenetic tree, it appears that *L. brevis* strains isolated from food and silage cluster on one branch of the tree, while *L. brevis* brewery isolates cluster on another. The *L. brevis* strains isolated from gut microbiota BDGP6 and ZLB004 as well as *L. brevis* strains NCTC13768 and 100D8 gather in a smaller clade.

### Pan/core-genome analysis

**Fig. 3:**
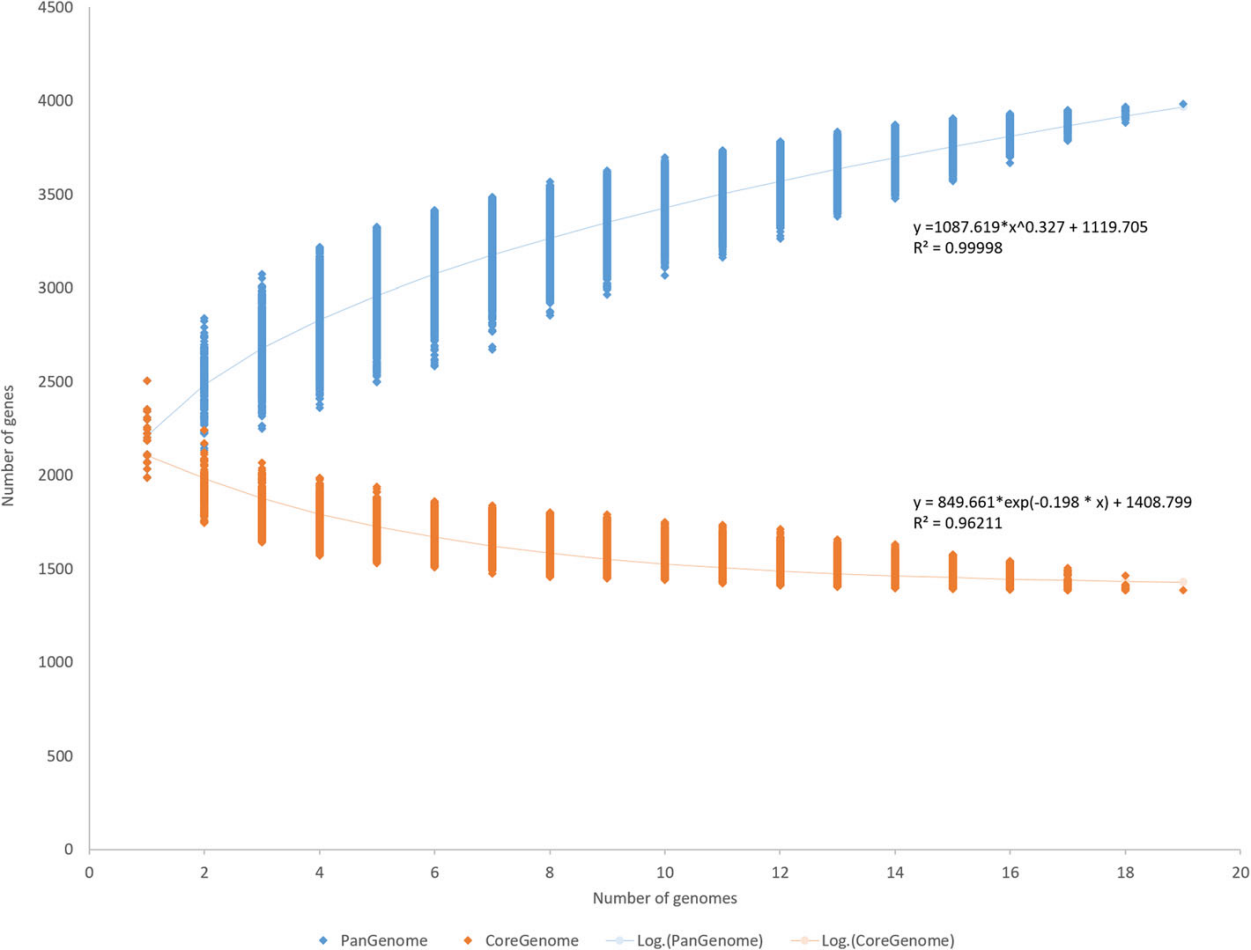
Pan- and core-genome of *L. brevis*. Accumulated number of new genes in the *L. brevis* pan-genome plotted against the number of new genomes added as well as accumulated number of genes attributed to the core-genome plotted against the number of genomes added. Deduced mathematical functions are also displayed on the graph

A pan-genome analysis was performed in order to determine the total number of distinct genes present on the combined chromosomal sequences of the analyzed *L. brevis* strains. The pan-genome curve displays an asymptotic trend, growing with an average rate of 136 genes per genome in the first nine iterations, then the number of new genes decreased leading to a total pan-genome content of 3968 genes (Fig. 3). The mathematical function displayed on the graph reveals an exponential value lower than 0.5 indicating that the pan-genome is in a closed state. The core genome was determined to encompass 1428 genes (Fig. 3). As an overall result, both analyses indicate a closed pan-genome for *L. brevis* species, while also indicating that a sufficient number of strains had been included to adequately describe the genetic repertoire of the *L. brevis* species.

### Comparative analysis of orthologous genes

**Fig. 4:**
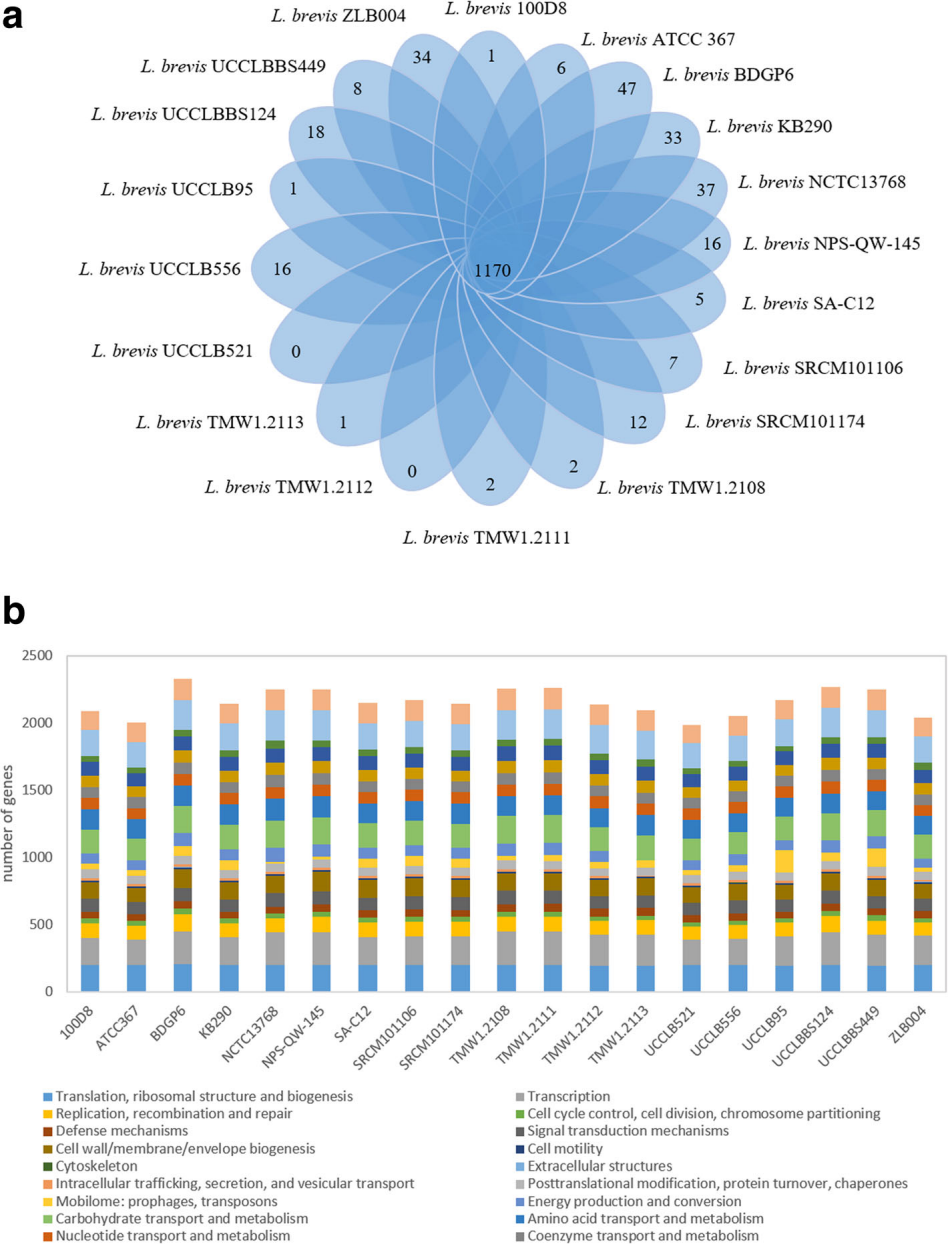
Comparative genomics of chromosomal orthologous proteins in *L. brevis.* **Panel a:** Venn diagram representing the orthologous and unique gene families of 19 *L. brevis* strains obtained by MCL clustering. **Panel b:** Cluster of Orthologous Groups (COG) classification of *L. brevis*. Histograms represent COG predictions for each of the following 16 *L. brevis* isolates: *L. brevis* 100D8, *L. brevis* ATCC 367, *L. brevis* BDGP6, *L. brevis* KB290, *L. brevis* NCTC13768, *L. brevis* NPS-QW-145, *L. brevis* SA-C12, *L. brevis* SRCM101106, *L. brevis* SRCM101174, *L. brevis* TMW 1.2108, *L. brevis* TMW 1.2111, *L. brevis* TMW 1.2112, *L. brevis* TMW 1.2113, *L. brevis* UCCLB521, *L. brevis* UCCLB556, *L. brevis* UCCLB95, *L. brevis* UCCLBBS124, *L. brevis* UCCLBBS449, *L. brevis* ZLB004

The comparative analysis used in this study was based on chromosomal sequences. The core genome of 1428 genes is divided in 1170 orthologous gene families (single copy) and 258 paralogous gene families (multi-copy). Unique gene families to each chromosome were also recorded and 246 unique gene families were identified across the nineteen *L. brevis* strains (Fig. 4a). Functional assignment efforts revealed that 75.2% of the unique gene families encoded proteins of unknown function (hypothetical proteins), while 4.5% encoded (pro)phage-related proteins. The remaining unique gene families encode proteins that could benefit the fitness of the strain such as CRISPR-Cas system (e.g. Type I-E CRISPR Cas system in *L. brevis* ZLB004), restriction-modification systems (e.g. Type I R/M system in *L. brevis* strain UCCLBBS124), or cell wall polysaccharide synthesis (e.g. genes predicted to encode glycosyltransferases and a polysaccharide polymerase only found in the *L. brevis* strain NPS-QW-145).

In order to further investigate the functionality and diversity encoded by the core and dispensable genomes, a Cluster of Orthologous Group (COG) analysis was employed. The genome content of the 19 selected *L. brevis* strains was classified into different groups depending on their function. More than 75% were predicted to be involved in housekeeping functions, vital for the strain to grow such as those participating in transcription or translation. Approximately 16% of the genes were assigned to COG groups with only a general function predicted or of unknown function (Fig. 4b).

### Evolution and adaptation to beer environment

**Fig. 5:**
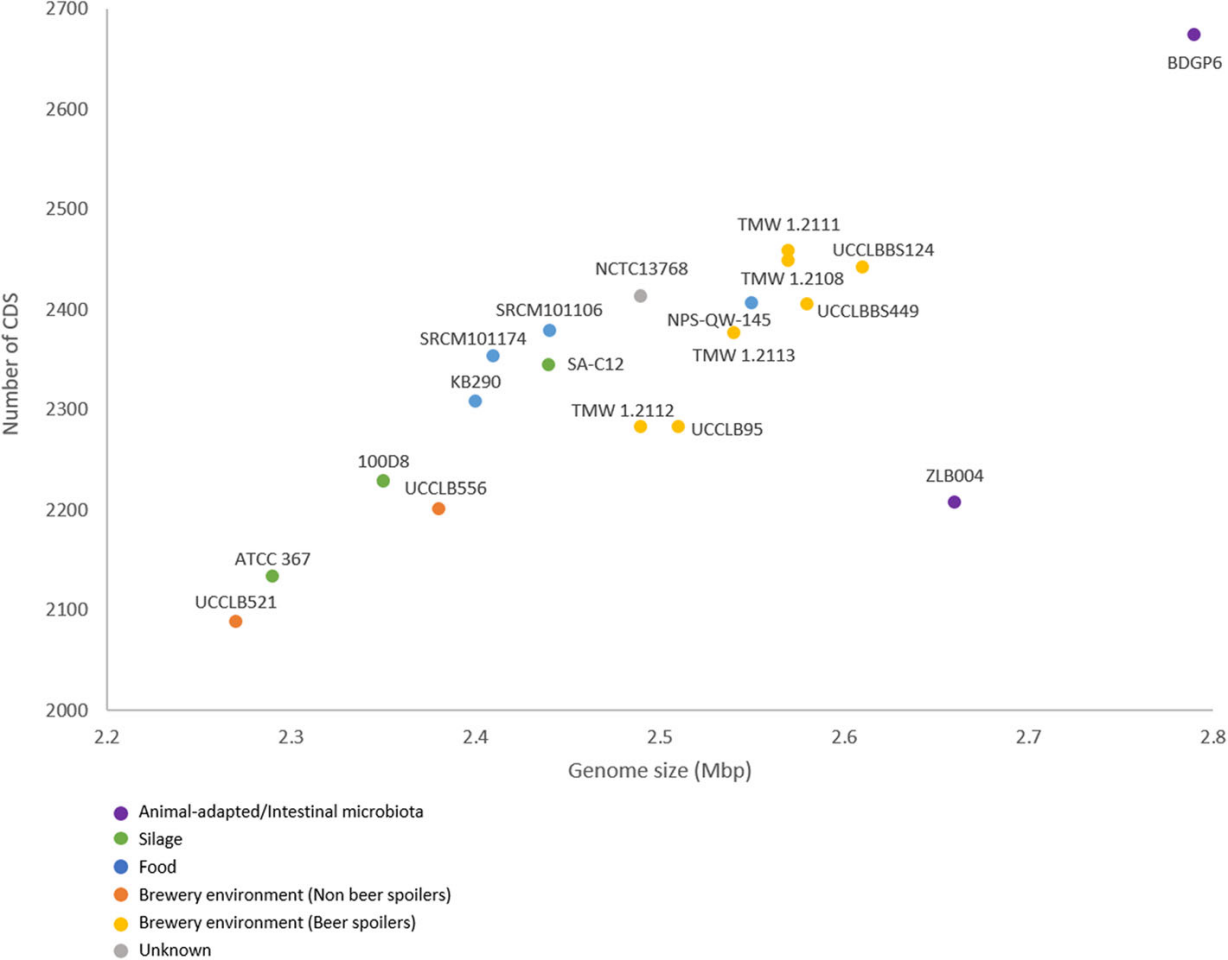
Association between chromosome size and CDS number in nineteen *L. brevis* complete chromosomal sequences

When plotting the number of CDSs as a function of genome size for the different *L. brevis* strains, the group exhibiting the largest genome size as well as the highest number of CDS are *L. brevis* strains isolated from beer and characterized as beer-spoilers as well as the *L. brevis* strain BDGP6 displaying the biggest CDS number. *L. brevis* strains known to be beer-spoilers possess an average of 2385 CDS, while those isolated from food, silage, animal’s gut and non-beer spoiling brewery isolates display an average of 2311 CDSs (Fig. 5). This observation suggests a link to adaptation to a new environment, i.e. the beer or brewery environment, which may have necessitated the acquisition of novel genes and corresponding functions in order to survive in this harsh environment. To understand if the beer-spoiling strains had acquired a specific set of genes or associated functions, genes that may putatively be associated to beer adaptation were first predicted to be those that would be present in the genomes of at least four beer spoiling strains (Table 5). From this analysis, 58 genes of interest were highlighted as well as 26 hypothetical proteins. Out of these 58 genes, approximately 21% encode proteins related to oxido-reduction reactions (Flavodoxin, oxidoreductases and short-chain dehydrogenases), 22% are linked to transcription (transcriptional regulators, RNA polymerase sigma-24 subunit ECF subfamily), 21% encode membrane and cell surface proteins and 14% are related to membrane transport (MFS transporter, permease, ABC transporters) (Table 5).

**Table 5:** List of genes identified in the chromosome sequence of at least four *L. brevis* beer spoiler strains. 26 genes coding for hypothetical proteins were also identified

COG category and protein function	*L. brevis* beer-spoiler strains						
	TMW1.2108	TMW1.2111	TMW1.2112	TMW1.2113	UCCLB95	UCCLBBS124	UCCLBBS449
	Energy production and conversion						
Flavodoxin	+	+	+	+	+	+	+
NADH-Flavin reductase	+	+	–	–	+	+	+
Oxidoreductase	+	+	+	+	+	+	+
NADPH:quinone reductase	+	+	–	–	+	+	+
FMN-dependent NADH-azoreductase	+	+	–	–	+	+	+
Nitrobenzoate reductase	+	+	–	–	+	+	+
	Amino acid transport and metabolism						
Shikimate dehydrogenase	+	+	+	+	+	+	+
Acetyltransferase GNAT family	+	+	–	–	+	+	+
Serine O-acetyltransferase EC	+	+	–	–	+	+	+
	Carbohydrate transport and metabolism						
MFS transporter	+	+	–	–	+	+	+
Alpha-glucosidase	+	+	+	+	+	+	+
lycoside hydrolase	+	+	–	–	–	+	+
Hydrolase	+	+	+	+	+	+	+
Transketolase	+	+	+	+	–	–	–
MFS transporter	+	+	+	–	+	+	–
PTS system2C IIA component 1	+	+	–	–	+	+	+
Putative integral membrane protein 1	+	+	–	–	+	+	+
PTS2C EIIB 1	+	+	–	–	+	+	+
PTS mannitol transporter subunit IIA	+	+	–	–	+	+	+
Putative oligogalacturonide transporter	+	+	+	+	+	–	+
	Coenzyme transport and metabolism						
6-pyruvoyl tetrahydropterin synthase	+	+	+	+	–	+	–
	Lipid transport and metabolism						
NADH peroxidase	+	+	+	+	+	+	+
Peroxidase	+	+	–	–	+	+	+
Citrate lyase	+	+	–	–	+	+	+
	Transcription						
Transcriptional regulator2C TetR family	+	+	–	–	+	+	+
Transcriptional regulator	+	+	+	+	+	+	+
Transcriptional regulator TetR family	+	+	–	–	+	+	+
Transcriptional regulator	+	+	+	+	+	+	+
Internalin-J	–	+	+	+	+	+	+
RNA polymerase sigma-24 subunit ECF subfamily	+	+	+	+	+	+	+
ECF-type sigma factor negative effector	+	+	+	+	+	+	+
Transcriptional regulator	+	+	+	+	+	+	+
Transcriptional regulator MarR family	+	+	–	–	+	+	+
Transcriptional regulator	+	+	–	–	+	+	+
Transcriptional regulator MarR family	+	+	–	–	+	+	+
Transcriptional regulator TetR	+	+	–	–	–	+	+
Transcriptional regulator ArsR family	+	+	–	–	+	+	+
	Cell wall/membrane/envelope biogenesis						
Membrane protein	+	+	–	+	–	+	–
Cell surface protein	+	+	+	+	–	–	–
Cell surface protein	+	+	–	–	–	+	+
Endo polygalacturonase	+	+	+	+	–	–	+
Glutamyl endopeptidase precursor	+	+	+	+	+	+	+
NLP-P60 protein	+	+	+	+	+	+	+
Short-chain dehydrogenase-oxidoreductase	+	+	–	–	+	+	+
	Inorganic ion transport and metabolism						
Permease	+	+	+	+	+	+	+
Permease	+	+	+	+	+	–	+
Na + −H+ antiporter	+	+	–	–	+	+	–
	General function prediction only						
NADPH-quinone reductase	+	+	+	+	+	+	+
Short-chain dehydrogenase-oxidoreductase	+	+	+	+	+	+	+
Short-chain dehydrogenase	+	+	+	+	+	+	–
Cell surface adherence protein	–	–	+	+	+	+	+
Mucus-binding protein LPXTG-motif cell wall anchor	+	+	+	+	+	–	+
	Function unknown						
Cell surface hydrolase	+	+	+	+	+	+	+
Membrane protein	+	+	+	+	–	+	+
Cell surface protein	+	+	–	–	+	+	+
	Defence mechanisms						
ABC transporter ATP-binding protein	+	+	+	+	+	+	+
ABC transporter permease	+	+	+	+	+	+	+
Prophage protein	+	+	+	+	–	–	+

+: gene present, −: gene absent

When exposed to beer, bacteria are subjected to various stresses, among them a low pH (3.8–4.7) and hop compounds [10]. When iso-α-acids enter the cell cytoplasm, they dissociate into hop anions and protons decreasing the intracellular pH [10]. Therefore, bacteria would have to adapt in order to regulate their internal pH in order to survive [28–30]. Furthermore, in beer the presence of ethanol (0.5–10% w/w) causes oxidative stress in bacteria, this results in the production of Reactive Oxygen Species (ROS) such as hydrogen peroxide and free radicals leading to cell damage [31, 32]. Despite the stress and harsh environment imposed by the beer environment, some bacteria have evolved and acclimatized to this new medium. It may thus be possible that some of the *L. brevis* strains acquired additional functions which allow them to grow and survive in beer and which has led to an increased genome size. The fact that 21% of these chromosomal genes encode proteins related to redox reactions is of interest and suggests a link between *L. brevis* beer-spoiler strains and oxidative stress response. Six of the 12 genes that encode functions relating to oxido-reduction reactions present in at least four beer-spoiler *L. brevis* strains are predicted to encode NADH oxidoreductases and short-chain dehydrogenases/reductases (SDRs). These proteins are part of the large family of NAD(P)(H)-dependent oxidoreductases and are believed to behave as scaffold proteins for an NAD(P)(H) redox sensor system [33]. In previous studies, the role of SDRs during oxidative stress was highlighted in species such as *Bacillus subtilis* where they are required for survival in severe ethanol stress [34], or in *Burkholderia pseudomallei* during salt stress [35].

Furthermore, 22% of the chromosomal genes that seem to be specifically associated with beer-spoiling *L. brevis* strains are linked to transcriptional regulation, suggesting that these regulators act on specific genes to control their expression and confer an advantage when present in beer. It would be interesting to study which genes are affected by these transcriptional regulators to assess the mechanisms employed to survive in this harsh environment. Of the *L. brevis* beer-spoiler specific chromosomal genes 21% encode membrane and cell surface proteins suggesting an adaptation to survive in the harsh beer environment. 14% encode proteins associated with membrane transport such as permeases and ABC transporters suggesting exchange between the strain and its environment and possibly a role in extrusion where the *L. brevis* isolate would expel protons or iso-α-acids in order to survive and thrive in beer, as has been described previously [10, 13, 15].

Interestingly some of the chromosomal genes identified among *L. brevis* beer-spoiler strains in this analysis had also been highlighted in a previous study as beer-spoilage diagnostic marker genes (DMG) [36]. These genes are predicted to code for an oligogalacturonide transporter, a short chain dehydrogenase and a RNA polymerase sigma factor ECF subfamily, which reinforces the hypothesis for their involvement in beer spoilage adaptation.

### The role of plasmids in adaptation to beer environment

Different proteins involved in beer spoilage have been identified on plasmids indicating the importance of plasmids for bacterial strains in beer spoilage. This might suggest a role for plasmid mobilization and transfer between bacterial strains throughout evolution to adapt to a new environment such as beer.

The nineteen analyzed *L. brevis* strains were predicted to harbour up to nine plasmids with strains *L. brevis* KB290 and *L. brevis* UCCLBBS449 exhibiting the largest plasmid complements of the assessed strains. The plasmid size ranges from 2.8 Kb to 107.0 Kb (Table 3). The number of plasmids and their size do not appear to be linked to the isolation source of the *L. brevis* strains (e.g. four plasmids for *L. brevis* SRCM101106 versus nine plasmids for *L. brevis* KB290, both isolated from fermented food) or to the beer spoilage ability of the isolate (two plasmids for *L. brevis* UCCLB95 versus nine plasmids for *L. brevis* UCCLBBS449 both characterised as beer-spoilers). Investigating analogies between plasmids among *L. brevis* beer-spoiler strains revealed that the plasmid content of *L. brevis* TMW1.2108 and *L. brevis* TMW1.2111 were very similar. Indeed, the six plasmids of *L. brevis* TMW1.2111 show at least 90% identity to seven of the eight plasmids contained by strain *L. brevis* TMW1.2108, with the exception of plasmid TMW1.2108–5. Similarly, *L. brevis* strains TMW1.2112 and TMW1.2113 present a close plasmid composition as the four plasmids of *L. brevis* TMW1.2113 are at least 90% identical to four out of five plasmids of *L. brevis* TMW1.2112 with the exception of plasmid TMW1.2112–1.

Out of the 38 plasmids shared between *L. brevis* beer-spoiler strains, only three plasmids seem to be unique, sharing less than 10% similarity with any other plasmid. These three plasmids were found in *L. brevis* UCCLBBS449 (UCCLBBS449_pF, UCCLBBS449_pH and UCCLBBS449_pI) and contain mostly genes coding for hypothetical proteins, replication proteins as well as genes coding for proteins involved in conjugation such as mobilization proteins and a relaxase.

Refined analysis of specific genes shared only between at least three *L. brevis* beer-spoiler strains, identified only twenty-five genes (Table 6). In this list of unique genes shared only between *L. brevis* beer-spoiler strains, the gene coding for the membrane protein HorC is noteworthy, as it is known to be involved in beer spoilage [14] and is present in all *L. brevis* beer-spoiler strains with the exception of *L. brevis* TMW1.2113.

**Table 6:** List of genes specifically only present in plasmid sequences of at least three *L. brevis* strains characterized as beer-spoilers

COG category and protein function	*L. brevis* beer-spoiler strains						
	TMW1.2108	TMW1.2111	TMW1.2112	TMW1.2113	UCCLB95	UCCLBBS124	UCCLBBS449
	Defence mechanisms						
Membrane protein HorC	+	+	+	–	+	+	+
	Cell wall biogenesis						
Lipopolysaccharide biosynthesis glycosyltransferase	+	+	+	+	–	+	+
Lipopolysaccharide biosynthesis glycosyltransferase	+	+	+	+	–	+	+
	Lipid transport and metabolism						
Phospholipid-glycerol acyltransferase	+	+	+	+	–	+	+
1-acyl-sn-glycerol-3-phosphate acyltransferase	+	+	+	+	–	+	+
Fatty acid-binding protein DegV	–	–	+	+	–	+	+
	Carbohydrate transport and metabolism						
Glycosyl transferase family 2	+	+	+	–	–	+	–
Enolase	+	+	–	–	–	–	+
MFS transporter	+	+	+	+	–	–	–
	Transcription						
Sigma-70 region 4 family protein	+	+	–	–	–	–	+
Transcriptional regulator TetR family	+	+	+	–	–	+	+
	Nucleotide transport and metabolism						
Cytosine deaminase	+	+	–	+	–	+	+
	Inorganic ion transport and metabolism						
CrcB-like protein	+	+	–	–	–	–	+
	Replication, recombination and repair						
Cytosine-specific methyltransferase	+	+	–	–	–	+	–
Initiator RepB protein	+	+	–	–	–	–	+
	Function unknown						
Hypothetical protein	+	+	–	–	–	–	+
Hypothetical protein	+	+	+	+	–	–	+
Hypothetical protein	+	+	+	+	–	–	–
Hypothetical protein	+	+	–	–	–	–	+
Hypothetical protein	+	+	–	–	–	+	–
Hypothetical protein	+	–	+	–	–	+	–
PemK family protein	+	+	–	–	–	+	–
	Mobilome						
Transposase	+	+	–	–	–	+	
Mobilization protein	+	+	–	–	–	–	+
Mobilization protein	+	+	–	–	–	–	+

+: gene present, −: gene absent

Interestingly, the gene encoding the ABC transporter HorA [13] and present in *L. brevis* beer-spoiler strains TMW1.2108, TMW1.2111, TMW1.2113, UCCLBBS124 and UCCLBBS449 does not figure in this list as a similar protein can be found in plasmid sequences of the *L. brevis* strains KB290, SRCM101106 isolated from fermented food and *L. brevis* UCCLB556 isolated from the brewery and characterized as a non-beer spoiler. Moreover, the transmembrane protein HitA [16] has been identified only in two of the *L. brevis* beer-spoiler strains UCCLBBS449 and TMW1.2112. These observations reinforce the statement that involvement of these genes in beer survival and spoilage is not always verified as they are not consistently present in beer-spoiler organisms nor are always corresponding to beer spoilage ability if present in a strain [15]. The list of genes present only in *L. brevis* beer-spoiler strains shows that strains *L. brevis* TMW1.2108 and TMW1.2111 possess more than 90% of these genes whereas *L. brevis* UCCLB95 only possesses one gene coding for the membrane transporter HorC (Table 6). The remainder of the *L. brevis* beer-spoiler strains carry approximately 50% of these particular genes (Table 6).

Out of these 25 unique genes shared among *L. brevis* beer-spoiler strains approximately 25% code for hypothetical proteins of unknown function. Meanwhile, ~ 30% of these genes appear to encode cell wall-associated proteins either as membrane transporters (MFS transporter, HorC) or as cell wall biosynthesis (lipopolysaccharide glycosyltransferases, acyltransferases). As mentioned above, a beer-spoiling strain would need to extrude toxic compounds using transporters and adapt its cell wall composition to survive the harsh beer environment. A smaller portion of these unique genes are linked to transcription regulation, replication or mobilome.

Interestingly, some of the plasmid-associated genes identified among *L. brevis* beer-spoiler strains in this analysis have also been highlighted previously as unique attributes of beer-spoiling strain plasmids [36]. The gene coding for the CrcB like-protein involved in ion transport was found on plasmid BSO 464–2 of the *L. brevis* beer-spoiler strain BSO 464 as well as a gene coding for enolase involved in glucose metabolism. A gene coding for cytosine deaminase is present in five out of the seven *L. brevis* beer-spoiler strains used in this analysis (Table 6) and was identified as a unique attribute on the plasmid pPECL-8 of the beer-spoiler *Pediococcus claussenii* ATCC BAA-344 [36]. Moreover, a plasmid-associated gene coding for a glycosyltransferase family 2 was highlighted in the analysis (Table 6), this protein was associated with excess β-glucan formation leading to a slimy ropy phenotype in the *L. brevis* beer-spoiler TMW1.2112 [37]. This slimy phenotype was observed in the *L. brevis* strain UCCLBBS124 first described in this study (Table 1), and the gene coding for the glycosyltransferase family 2 was identified on one of its plasmid UCCLBBS124_pB.

This overall examination of plasmid-associated genes shows the importance of extrachromosomal DNA in beer spoilage adaptation and opens new possibilities for understanding the beer spoilage process with an updated list of potential proteins of interest only present in *L. brevis* beer-spoiler strains.

## Conclusions

The isolation and genome sequencing of six *L. brevis* strains combined with thirteen additional, publicly available *L. brevis* genomes allowed a comparative genome analysis of the *L. brevis* species. The deduced pan-genome of these *L. brevis* isolates appears to be in a closed state, indicating that the representatives used in this study are sufficient to describe the genetic diversity of the taxon. Throughout evolution, it appears that *L. brevis* strains specified and differentiated one from another by acquiring plasmids and prophages, despite for the presence of CRISPR-Cas and R/M systems which may have limited such foreign DNA invasion events. These latter systems are of relevance for future functional investigations that may necessitate the development of DNA transfer and/or mutagenesis tools. *L. brevis* strains represent a significant threat for the brewing industry being the most common cause of beer spoilage; however, this spoiling ability is strain specific. The comparative genome analysis performed here highlights that *L. brevis* strains with the ability to grow in beer possess a higher number of CDSs in their overall chromosomal sequences. This observation suggests a link to evolution and adaptation to beer in which the strain would have acquired novel genes and functions in order to adapt and survive in the harsh environment that beer represents. The role(s) of the “acquired” or beer-specific CDSs revealed that almost a quarter of these are linked to oxido-reduction reactions, possibly playing a role in the response to oxidative stress. Another 22% are linked to transcription regulation, 21% encode cell surface proteins while 14% are encoding membrane transport related proteins and possibly associated to harmful compound extrusion encountered by the *L. brevis* strains when surviving and growing in beer. Additional genetic diversification of these *L. brevis* strains is expected to have occurred through plasmid acquisition that also likely contributes to beer adaptation. The plasmid content analysis of the different *L. brevis* beer-spoiler strains highlighted the presence of unique proteins shared among these strains. These proteins are mostly hypothetical proteins while approximately 30% are linked to membrane transport, and cell-wall synthesis. These observations demonstrate the complexity of microorganisms’ beer spoilage ability and suggests that adaptation of the *L. brevis* strain to beer is a complex process, not due to the action of only one specific gene, but more likely the intervention of a complex, multi-factorial response.

## Methods

### Isolation of *L. brevis* strains

Five distinct *L. brevis* strains (UCCLB521, UCCLB556, UCCLB95, UCCLBBS124 and UCCLBBS449) were isolated from the brewing environment, while SA-C12 had previously been isolated from silage [22]. The strains were characterized by evaluating their plasmid content as well as growth curve profiles in MRS broth or in beer at 30 °C. Plasmids were isolated after overnight growth of the *L. brevis* strains in MRS broth at 30 °C, cells were pelleted by centrifugation for 10 min at 5000 rpm followed by lysozyme treatment (30 mg/mL lysozyme in TE + 25% sucrose) at 37 °C for 30 min. Plasmids were extracted using the GeneJET Plasmid Miniprep Kit (Thermo Scientific™). Plasmid profiles of the different *L. brevis* isolates were analyzed using a 1% agarose gel. Growth curve profiles in MRS broth or in beer were performed at 30 °C by hourly OD_600nm_ measurements for a period of 55 h. Moreover, colony morphology was recorded following growth on MRS agar plate at 30 °C.

### Sequencing and annotation

*L. brevis* strains were streaked on MRS agar plates and grown at 30 °C for 24 h. For each *L. brevis* strain, a single colony was inoculated into MRS broth and grown overnight at 30 °C. Cells were pelleted by centrifugation at 5000 rpm for 10 min. The supernatant was removed and the pelleted cells were frozen at − 20 °C prior sending for sequencing. Sequencing was performed using the PacBio SMRT next generation sequencing technology (performed by GATC Biotech, Germany). De novo genome assemblies were performed using the Pacific Biosciences SMRT Portal analysis platform. Open Reading Frame (ORF) or coding sequence (CDS) prediction was performed using Prodigal prediction software [38] and confirmed using BLASTX alignments [39]. Automatic annotations were refined using Artemis v16.0.0 where ORF predictions were manually checked, start codons adjusted and pseudogenes identified. Transfer RNA (tRNA) genes were predicted using tRNA-scan-SE v2.0 [40], while ribosomal RNA (rRNA) genes were identified using RNAmmer v1.2 [41]. The sixteen *L. brevis* genomes obtained from NCBI were re-annotated as described above in order to treat identically all sequenced genomes used in this study.

### Methylome analysis

Following de novo genome assembly, the RS_Modification_and_Motif_Analysis.1 protocol of the SMRT Analysis portal was employed for base modification and methylated motif detection. This analysis was performed on *L. brevis* strains sequenced, assembled and annotated as part of this study. Methylation motifs with a score equal or higher than 40 (corresponding to a *P*-value of < 0.0001) were considered specific and used for further analysis. ORFs of genomes were investigated for the presence of restriction/modification systems using the BLASTP alignment function of the REBASE database [42] (cut-off E-value of 0.0001; with at least 30% similarity over at least 80% of the sequence length). A comparative genome analysis was employed to associate the presence of R/M system-encoding genes with the presence of methylation motif(s).

### Comparative genomics

All protein sequence comparisons were performed using all-against-all, bi-directional BLAST alignments [39]. An alignment cut-off value of E-value 0.0001, and a similarity cut-off level of at least 30% amino acid identity across 80% of the sequence length was used. Results were analyzed with the Markov Clustering Algorithm (MCL) [43] and proteins encoded were categorized in predicted functional groups based on COG (Clusters of Orthologous Groups) assignments [44].

### Phylogenetic analysis

The supertree was prepared using the BLAST-based comparative approach described above in order to identify chromosomal orthologous proteins. The set of chromosomal orthologous proteins was concatenated for each strain and an ungapped alignment was performed using MUSCLE v3.8.31 [45]. The phylogenetic tree was computed using the maximum-likelihood method in PhyML v3.0 and bootstrapped employing 1000 replicates [46]. The final tree was visualized using MEGA7. A tree based on 16 S rRNA genes was constructed using clustalw and visualized via ITOL (Interactive Tree Of Life) [47]. The chromosome sequence of *Enterococcus faecalis* V583 (Accession: AE016830) was included as an outgroup.

### Pan/core-genome analysis

The pan-core genome analysis of the above-mentioned 19 *L. brevis* chromosomal sequences, was performed using PGAP v1.0 [48]. ORF content for each chromosome is classified in functional gene clusters using the Gene Family method. From this analysis a pan/core genome profile was generated.

### Genome accession numbers

*L. brevis* 100D8: CP015338, *L. brevis* ATCC 367: CP000416, *L. brevis* BDGP6: CP024635, *L. brevis* KB290: AP012167, *L. brevis* NCTC13768: LS483405, *L. brevis* NPS-QW-145: CP015398, *L. brevis* SA-C12: CP031185, *L. brevis* SA-C12_pA: CP031186, *L. brevis* SA-C12_pB: CP031187, *L. brevis* SRCM101106: CP021674, *L. brevis* SRCM101174: CP021479, *L. brevis* TMW 1.2108: CP019734, *L. brevis* TMW 1.2111: CP019743, *L. brevis* TMW 1.2112: CP016797, *L. brevis* TMW 1.2113: CP019750, *L. brevis* UCCLB521: CP031208, *L. brevis* UCCLB521_pA: CP031209, *L. brevis* UCCLB521_pB: CP031210, *L. brevis* UCCLB521_pC: CP031211, *L. brevis* UCCLB521_pD: CP031212, *L. brevis* UCCLB521_pE: CP031213, *L. brevis* UCCLB556: CP031174, *L. brevis* UCCLB556_pA: CP031175, *L. brevis* UCCLB556_pB: CP031176, *L. brevis* UCCLB556_pC: CP031177, *L. brevis* UCCLB556_pD: CP031178, *L. brevis* UCCLB556_pE: CP031179, *L. brevis* UCCLB556_pF: CP031180, *L. brevis* UCCLB556_pG: CP031181, *L. brevis* UCCLB95: CP031182, *L. brevis* UCCLB95_pA: CP031183, *L. brevis* UCCLB95_pB: CP031184, *L. brevis* UCCLBBS124: CP031169, *L. brevis* UCCLBBS124_pA: CP031170, *L. brevis* UCCLBBS124_pB: CP031171, *L. brevis* UCCLBBS124_pC: CP031172, *L. brevis* UCCLBBS124_pD: CP031173, *L. brevis* UCCLBBS449: CP031198, *L. brevis* UCCLBBS449_pA: CP031199, *L. brevis* UCCLBBS449_pB: CP031200, *L. brevis* UCCLBBS449_pC: CP031201, *L. brevis* UCCLBBS449_pD: CP031202, *L. brevis* UCCLBBS449_pE: CP031203, *L. brevis* UCCLBBS449_pF: CP031204, *L. brevis* UCCLBBS449_pG: CP031205, *L. brevis* UCCLBBS449_pH: CP031206, *L. brevis* UCCLBBS449_pI: CP031207, *L. brevis* ZLB004: CP021456 and *Enterococcus faecalis* V583: AE016830.

## Abbreviations

BLAST: Basic Alignment Search Tool
CDS: Coding Sequence
COG: Cluster of Orthologous Group
CRISPR: Clustered Regularly Interspaced Short Palindromic Repeats
DMG: Diagnostic Marker Genes
ITOL: Interactive Tree Of Life
LAB: Lactic Acid Bacteria
MCL: Markov Clustering Algorithm
ORF: Open Reading Frame
QPS: Qualified Presumption of Safety
R/M: Restriction/Modification
ROS: Reactive Oxygen Species
SDR: Short-chain Dehydrogenase/Reductase
SMRT: Single-Molecule-Real-Time sequencing
